# Enhanced Bayesian Spline Regression Approach for Modelling Trends in Infections Caused by Pathogens Commonly Transmitted Through Food

**DOI:** 10.15212/zoonoses-2025-0030

**Published:** 2026-03-10

**Authors:** Daniel L. Weller, Samantha Sevilla, Ethan Hetrick, Erica Billig Rose, Joshua Forstedt, Jason Caravas, Logan C. Ray, Daniel C. Payne, Molly K. Steele, Robert M. Hoekstra, Beau B. Bruce

**Affiliations:** 1 Enteric Diseases Epidemiology Branch, Division of Foodborne, Waterborne, and Environmental Diseases, National Center for Emerging and Zoonotic Infectious Diseases, Centers for Disease Control and Prevention, Atlanta, GA, USA; 2 Leidos Inc., Reston, VA 20190, USA; 3 Office of Advanced Molecular Detection, Division of Infectious Disease Readiness and Innovation, National Center for Emerging and Zoonotic Infectious Diseases, Centers for Disease Control and Prevention, Atlanta, GA, USA; 4 Current address: Division of Infectious Diseases, Cincinnati Children’s Hospital Medical Center, Cincinnati, OH, USA; 5 Current address: Department of Pediatrics, University of Cincinnati College of Medicine, Cincinnati, OH, USA

**Keywords:** FoodNet, Bayesian regression, splines, nowcasting, foodborne disease, foodborne diseases active surveillance network

## Abstract

**Background:**

The Foodborne Diseases Active Surveillance Network (FoodNet) monitors illnesses caused by enteric pathogens at 10 U.S. sites to track progress toward federal disease reduction goals. The original trends frequentist model was developed to overcome statistical complications associated with FoodNet’s catchment expansion during 1996–2004 but did not account for site-specific trends and treated year as a categorical variable. It was therefore sensitive to noise and single-year events, and shaped by trends in more populous sites.

**Methods:**

This paper describes an enhanced Bayesian model that overcomes these challenges by treating year as continuous, including an interaction between year and site, and using splines to better capture non-linear trends.

**Results:**

The enhanced model generates improved uncertainty estimates, can estimate incidence for years not represented by the surveillance dataset, and is dataset-agnostic. By publishing a pipeline for running the enhanced model, it can be readily adapted (i) for modeling trends in individual sites or among specific populations; (ii) for use with alternative datasets; and (iii) to include additional covariates.

**Conclusions:**

FoodNet generates the most up-to-date and highest-resolution enteric disease surveillance data in the United States. Publishing the pipeline for implementing this model improves methodologic transparency, and makes FoodNet data more accessible and available for guiding public health action in the near-real time.

## Brief statement

The Foodborne Diseases Active Surveillance Network (FoodNet) monitors illnesses caused by enteric and food-borne pathogens at 10 U.S. sites. FoodNet data are used in a trends model that tracks progress toward federal disease reduction goals. This paper describes enhancements to this model that are designed to (i) reduce the impact of noise or single-year events, such as foodborne outbreaks, on incidence estimates; (ii) reduce bias toward trends in more populous sites; (iii) improve uncertainty estimation; and (iv) support nowcasting of incidence for years not fully represented in the surveillance dataset. These improvements increase the reliability and interpretability of disease estimates, enabling more effective public health action. Publishing a transparent and reproducible pipeline for running the enhanced model also improves methodological transparency and makes FoodNet data more accessible for diverse applications.

## Graphical abstract

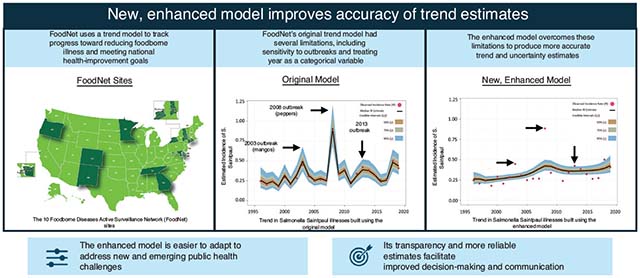

## INTRODUCTION

The World Health Organization estimates that approximately 600 million illnesses caused by 31 foodborne hazards occurred in 2010, including approximately 95.6 million *Campylobacter*, 78.7 million non-typhoidal *Salmonella* (NTS), 51.0 million *Shigella*, 1.2 million Shiga toxin-producing *Escherichia coli* (STEC), and 14,169 *Listeria monocytogenes* infections [1]. Using data from 2000 to 2008, Scallan et al. estimated that *Campylobacter*, NTS, *Shigella*, and STEC were among the leading causes of domestically acquired foodborne illness in the United States and attributed a smaller, but non-negligible, disease burden to *Cyclospora, L. monocytogenes*, *Vibrio*, and *Yersinia* [2]. *L. monocytogenes* was estimated to cause approximately 1,600 illnesses annually, resulting in approximately 1,500 hospitalizations and 255 deaths [2]. More recently, a 2025 paper estimated that 1.87 million *Campylobacter*, 1,250 *Listeria*, 1.28 million *Salmonella*, and 357,000 STEC domestically acquired, foodborne infections occur in the United States each year resulting in 197, 172, 238, and 66 deaths, respectively [3]. Recognizing the burden of foodborne illness in the United States, the U.S. federal government established disease reduction goals for domestically acquired illnesses caused by a subset of pathogens commonly transmitted through food [4].

Reducing enteric disease incidence requires an up-to-date understanding of illness epidemiology and trends. The U.S. Centers for Disease Control and Prevention, U.S. Food and Drug Administration (FDA), and U.S. Department of Agriculture’s Food Safety Inspection Service (FSIS), in collaboration with 10 state health departments, established the Foodborne Diseases Active Surveillance Network (FoodNet) to support these efforts. FoodNet is a sentinel surveillance program that provides regulatory and public health agencies, industry, and consumer groups with accurate information on incidence and trends in these illnesses. When FoodNet was founded, the catchment area represented 14.3 million people, and included Minnesota (MN), Oregon (OR), and select counties in California (CA), Connecticut (CT), and Georgia (GA). The catchment area expanded consistently from 1996 through 2004 to include all of CT, GA, Maryland (MD), MN, New Mexico (NM), OR, and Tennessee (TN), and select counties in CA (N = 3), Colorado (CO; N = 7), and New York (NY; N = 34) [5]. During 2004–2022, the catchment area remained constant but expanded again in 2023 to include the remainder of CO. During 2004–2022, the FoodNet catchment area represented approximately 15% of the U.S. population; as of 2023, the catchment area represented approximately 16% [5].

Part of FoodNet’s mission is to monitor trends in illnesses caused by eight pathogens commonly transmitted through food (*Campylobacter, Cyclospora, Listeria, Salmonella, Shigella*, STEC, *Vibrio*, and *Yersinia*) as well as pediatric hemolytic uremic syndrome. FoodNet’s continued growth from 1996 through 2004, coupled with substantial variation in population and incidence between sites, meant identifying a suitable approach for modeling trends was challenging [6]. As a result, analyses conducted before 2001 used data from the 1996 catchment and did not leverage data from sites added between 1996 and 2001. To effectively utilize data from the entire FoodNet catchment area, a log-linear negative binomial regression trend model was developed by Henao et al. [6]. This frequentist model overcame statistical challenges with FoodNet’s expansion during 1996–2004 by treating year as a categorical variable and estimating change in incidence over time by comparing incidence in a given year to a pre-specified reference year [6]. From 2001 to 2021, this model was used to track progress toward federal disease reduction efforts [7,8].

**FIGURE 1 |:**
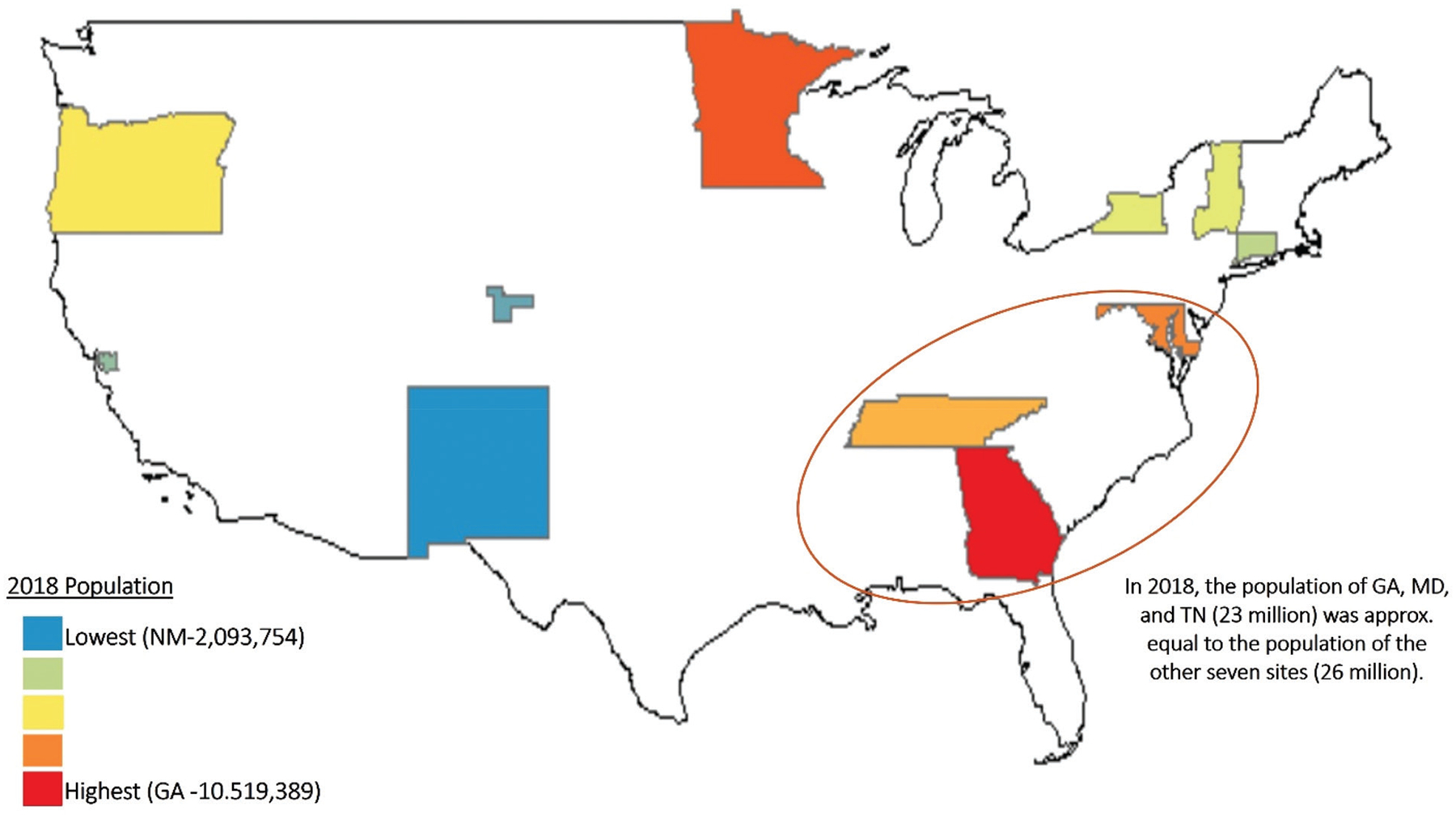
The current FoodNet model is shaped by trends in the populous southeastern sites.

The FoodNet trends model was developed to capture long-term illness incidence trends in the FoodNet catchment. While the model developed by Henao et al., henceforth the original model, leveraged FoodNet data collected from 1996 onwards from all 10 FoodNet sites, the model had several limitations [6]. Because the FoodNet trends model is used to track progress toward federal disease reduction goals annually [4], the Centers for Disease Control and Prevention (CDC) determined an enhanced model was needed that leveraged recent computational and statistical advancements to overcome these limitations. For example, the original model was biased toward trends in more populous sites (Fig 1). The enhanced model addresses this issue by calculating separate trends for each FoodNet site and combining the posterior estimates. Additionally, by treating year as continuous rather than categorical and using splines, the enhanced model reduces sensitivity to noise, missing data, and one-off events, such as single-year outbreaks, which can distort long-term trends. The present study describes this enhanced model. Although the enhanced model has been used in FoodNet annual reports since 2022 [5,9,10], this is the first peer-reviewed manuscript to describe it and directly compare the original and enhanced models. Additionally, by publishing a fully reproducible pipeline and code, along with the Nextflow pipeline for implementation, this work enhances the transparency and accessibility of FoodNet trend modeling. This enables its application beyond FoodNet and expands its utility for public health surveillance, making it a versatile tool that can be adapted for new and innovative uses across different datasets or population groups.

## METHODS

### Data

FoodNet is a collaboration among 10 state health departments, the U.S. CDC, FDA, and FSIS. FoodNet conducted population-based, active surveillance for laboratory-confirmed infections caused by *Campylobacter, Cryptosporidium, Cyclospora, L. monocytogenes, Salmonella*, *Shigella*, STEC, *Vibrio*, and *Yersinia*, as well as pediatric hemolytic uremic syndrome. FoodNet stopped collecting data for *Cryptosporidium* infections in 2017, and for *Campylobacter* infections that were not culture confirmed in 2023. FoodNet made collection of data optional for all diseases except Salmonella and STEC infection in 2025.

For each illness caused by a FoodNet-monitored pathogen, demographic, symptom, and other epidemiologic data are collected. Cases were considered domestically acquired if the ill person did not report international travel or had an unknown travel history during the ≤30 days before *Listeria*, *Salmonella* Typhi, and *Salmonella* Paratyphi symptom onset, ≤14 days before cyclosporiasis onset, and ≤7 days before symptom onset for other infections. FoodNet began collecting data on illnesses diagnosed using culture-independent diagnostic tests in 2012. FoodNet data collected during 1996–2019 were used to develop and compare the models reported here.

### Original model parameterization

The original FoodNet model used negative binomial regression implemented in SAS to model trends in disease incidence using surveillance data aggregated to the county-year level [6] (Table 1). For the study reported here, this SAS code (SAS Institute Inc. Cary, NC, USA) was adapted to run in R. The results reported here were run using R version 4.4.0 (R Foundation for Statistical Computing, Vienna, Austria). County-year is a model-specific term that refers to counties within each site that entered the FoodNet catchment concomitantly. For example, the two CA counties that were part of the initial 1996 FoodNet catchment had a county-year value of CA_1996, while the CA county that joined FoodNet in 2000 had a value of CA_2000. Not all counties transmitted data for all FoodNet-monitored pathogens when they joined the FoodNet catchment. For example, some counties began transmitting data on bacterial illnesses to CDC FoodNet when they enrolled but began transmitting data on parasitic illnesses later. Different county-year values were therefore used when modeling trends for bacterial and parasitic illnesses using the original model.

The original model used a log link function, and a log offset for population. The outcome was incidence per 100,000 persons and the covariates were the year the infection occurred and county-year. Due to variation in incidence between sites and an excess of zeros in the surveillance data (i.e., years where no cases of a given illness occurred in a given jurisdiction), a negative binomial instead of a Poisson distribution was used. Year was treated as a categorical variable with the reference set to a relevant comparison period [6]. Change in incidence relative to this reference was calculated for years of interest (e.g., most recent year of data).

Selecting an appropriate reference year for tracking trends was challenging due to FoodNet’s expansion between 1996 and 2004, and because relevant baselines depend on the specific analytic question being addressed by the comparison. For example, progress toward the Healthy People 2030 foodborne disease reduction goals is monitored using average incidence during 2016–2018 as the baseline [4]. Other analyses used the first year of FoodNet surveillance [i.e., 1996; [11,12]], the first three years after FoodNet’s catchment stabilized [i.e., 2004–2006; [13]], and average incidence for the 3 years preceding the year of interest [7,14].

### Enhanced model parameterization

Models built using different distributions (e.g., Poisson, zero-inflated) and model parameterizations (e.g., spline of year without a site–year interaction) were considered. The top-performing models across all pathogens regardless of distribution and scale of data

1. used penalized thin plate regression splines to model the effect of year;
2. treated year as a continuous variable; and
3. incorporated a year–site interaction.

Models fit using county-level data performed comparably to but took an order of magnitude longer to run than site-level models, and the negative binomial distribution performed slightly better than models fit with other distributions. Thus, the final version of the enhanced model was a Bayesian negative binomial model implemented in R with a log link function and a log offset for the population of each site each year (Table 1). Data were aggregated to the site level, and illness count was modeled as a function of site and year, which is treated as a continuous variable. The new model allows for the estimation of separate, site-specific trends using penalized thin plate splines. Thin plate splines were selected because they (i) provide computationally efficient, stable, and optimal low-rank approximations, (ii) avoid issues related to knot placement through truncated Eigen-decompositions, and (iii) allow model selection using methods dependent on model nesting [15]. The priors and the value of the basis function used to represent the smooth term for the thin plate regression splines were set to the package default. For the fixed effects, an improper flat prior was used. For the splines, a half Student-t prior was used with the scale parameter dependent on the standard deviation of the transformed response.

The enhanced model was implemented with 6 chains and 10,001 iterations using the brms package [version 2.20.1; [16–18]]. Splines were implemented using the s() function with the *by* parameter set to site. The new model generated a posterior predictive distribution of estimated mean log illness counts for each site in each year. We obtained samples from this distribution using the add_linpred_draws function in the tidybayes package [version 3.0.7; [19]] and exponentiated them. Illness estimates across sites were then summed for each draw-year combination to generate a distribution of illness estimates for the entire FoodNet catchment population for each year during the study period. In Bayesian statistics, credible parameters are described by probability distributions. However, for comparability with frequentist statistics, a point estimate can be drawn from this distribution [e.g., median, maximum a posteriori estimate (MAP)] and a range of values that represent a given percentage (e.g., 89%, 95%) of the distribution’s probability mass can be identified to produce a credible interval (CrI) analogous to a confidence interval (CI). For the Bayesian model described here, MAP and median incidence estimates, and the highest density interval and equal-tailed 95% CrI were calculated for each year for each site and the entire FoodNet catchment. Median incidence and 95% CrI estimates were converted to incidence per 100,000 by using a given site’s population or the FoodNet catchment population. To determine if incidence increased, decreased, or stayed the same, incidence estimates were compared to average incidence during one or more reference periods. For example, in the 2024 FoodNet annual report, incidence estimates for 2023 were compared with average incidence estimates for 2016–2018, which is the baseline period used to track progress toward federal disease reduction goals.

A Nextflow (version 24.04.2) pipeline was developed to containerize the implementation of the enhanced model. This pipeline is designed to work with both raw and cleaned FoodNet and non-FoodNet data. To improve accessibility for non-technical users, it employs a series of sequential prompts that guide users through the necessary decisions for data cleaning and code execution. The pipeline offers significant flexibility, maximizing the utility of the enhanced model beyond its current FoodNet applications. The source R code, pipeline, and documentation can be accessed at https://github.com/CDCgov/FoodNetTrends.

### Comparison of original and enhanced models model performance

**FIGURE 2 |:**
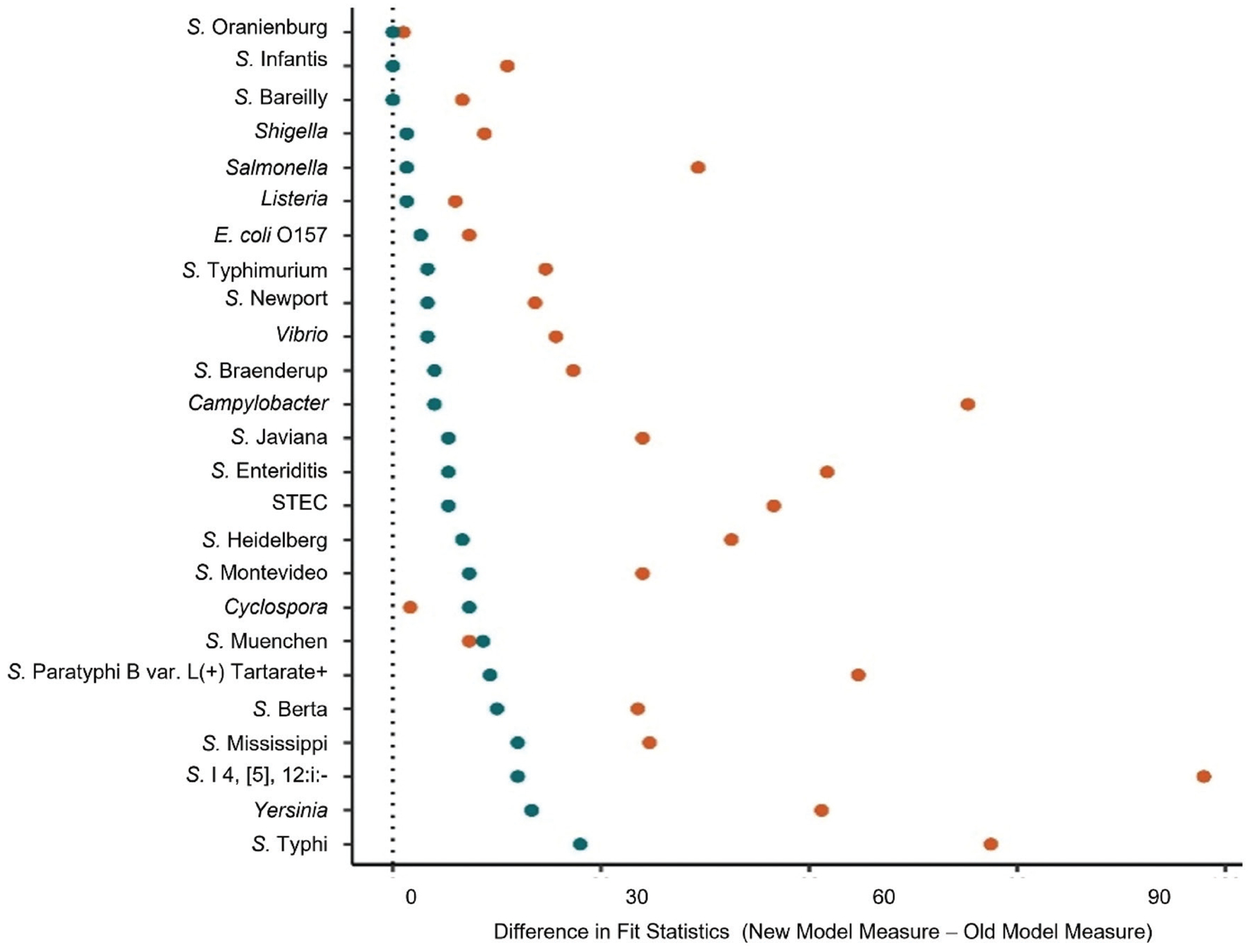
Improved fit of the new Bayesian splines model compared with a Bayesian version of the previous frequentist model. Fit was quantified using two statistical measures: estimated expected log pointwise predictive density (ELPD; **orange**) and R^2^ (**blue**). The difference was calculated between the new and old models for each measure. Results are shown for all pathogens tracked by FoodNet, including Shiga toxin-producing *E. coli* (STEC) overall and for *E. coli* O157:H7, specifically, as well as 16 *Salmonella* serotypes. Values above 0 (the dotted line) indicate that the new model fit the data better than the previous model; values below 0 would indicate the opposite. No values fell below 0. Note differences in R^2^ were multiplied by 100.

Separate models were implemented for each of the eight diseases monitored by FoodNet through 2019 (i.e., *Campylobacter*, *Cyclospora*, *Listeria*, *Salmonella*, STEC, *Shigella*, *Vibrio*, and *Yersinia* infection) as well as STEC O157, non-O157 STEC, and the most frequently reported *Salmonella* serotypes during 2016–2018 (see Fig 2).

To ensure comparability in model outputs, a Bayesian version of the original frequentist model was implemented in R using the brms package and an improper flat prior for the fixed effects [16–18]. A second, modified Bayesian version of the original model was implemented to facilitate comparison with the enhanced model (Table 1). This modified model used data aggregated at the site instead of county-year level and included a fixed effect of site instead of county-year [15,20–22]. Similarly, in addition to the enhanced model described above, a modified enhanced model was also implemented to facilitate comparison with the original model. The modified enhanced model used data aggregated at the county-year level, instead of site-level, and included an additional covariate for county-year. Frequentist versions of the original, the modified original, enhanced, and modified enhanced models were implemented using the mgcv package [15,20–22].

Estimates from the paired frequentist and Bayesian models, and from the paired county-year and site-level models, were compared to ensure consistency across frameworks and spatial scales for the original and enhanced models. The original and enhanced models were compared visually using the ggplot2 package (version 3.5.1; [23]) and quantitatively using root mean squared error (RMSE), adjusted R^2^, and expected log pointwise predictive density (ELPD), which were calculated from in-sample fits using the performance package [24].

## RESULTS AND DISCUSSION

Estimates from the frequentist and Bayesian versions of the original and enhanced models were similar; this is consistent with past studies that compared estimates from frequentist analyses and Bayesian analyses, including analyses with weakly informed priors [25–28]. The enhanced model and Bayesian version of the original model that were fit using data aggregated to the county-year performed comparably to, and generated similar catchment-level incidence estimates as, models fit using site-level data. However, the county-year models took over an order of magnitude longer than the site-level models to finish. The computational costs for Markov chain Monte Carlo (MCMC)-based Bayesian analyses are well established in the literature, and generally increase as model complexity, spatial resolution, and data size increase [27]. Due to computational concerns and the comparable performance between the county-year and site-level models, the site-level model was selected as the final form of the enhanced model and has been used by FoodNet to track trends in foodborne and enteric disease since 2022 [5,9,10]. Because the FoodNet trends model was designed to support catchment-wide surveillance and track progress toward national federal disease reduction goals, site-level analyses are sufficient for its current use. However, we recognize that site-level aggregation can mask within-site variability. Indeed, a recently published paper adapted the FoodNet model to compare salmonellosis trends within Virginia counties, highlighting this variability and the potential benefits of sub-site analyses [29]. Future efforts to develop county-level models may therefore enhance the model’s utility for guiding localized interventions, support decision-making in sites with substantial heterogeneity, and better leverage within-site variability when generating catchment-wide estimates.

Across pathogens, the enhanced model outperformed the Bayesian version of the original model. All performance metrics (R^2^, ELPD, RMSE) reported here were calculated from in-sample fits, as the goal of this study was to evaluate methodological improvements relative to the original model rather than to assess out-of-sample forecasting accuracy. The greatest improvement in performance was for *S*. Typhi [R^2^ Difference (ΔR^2^) = 0.27] and *Yersinia* (ΔR^2^ = 0.20; Fig 2). After *Yersinia*, ΔR^2^ ranged between 0.02 and 0.11 for the pathogen models, and between 0.00 and 0.27 for the *Salmonella* serotype models with improvements >0.10 for eight serotypes (Fig 2). While there was no improvement in R^2^ for the non-O157 STEC and *Salmonella* Bareilly, Infantis, and Oranienburg models, the enhanced models for these pathogens showed improvements in ELPD and RMSE compared to the original models (Fig 2). Overall, the consistent improvements across pathogens indicate that the enhanced model more accurately captures long-term trends compared to the original model.

### The enhanced model generates improved uncertainty estimates

**FIGURE 3 |:**
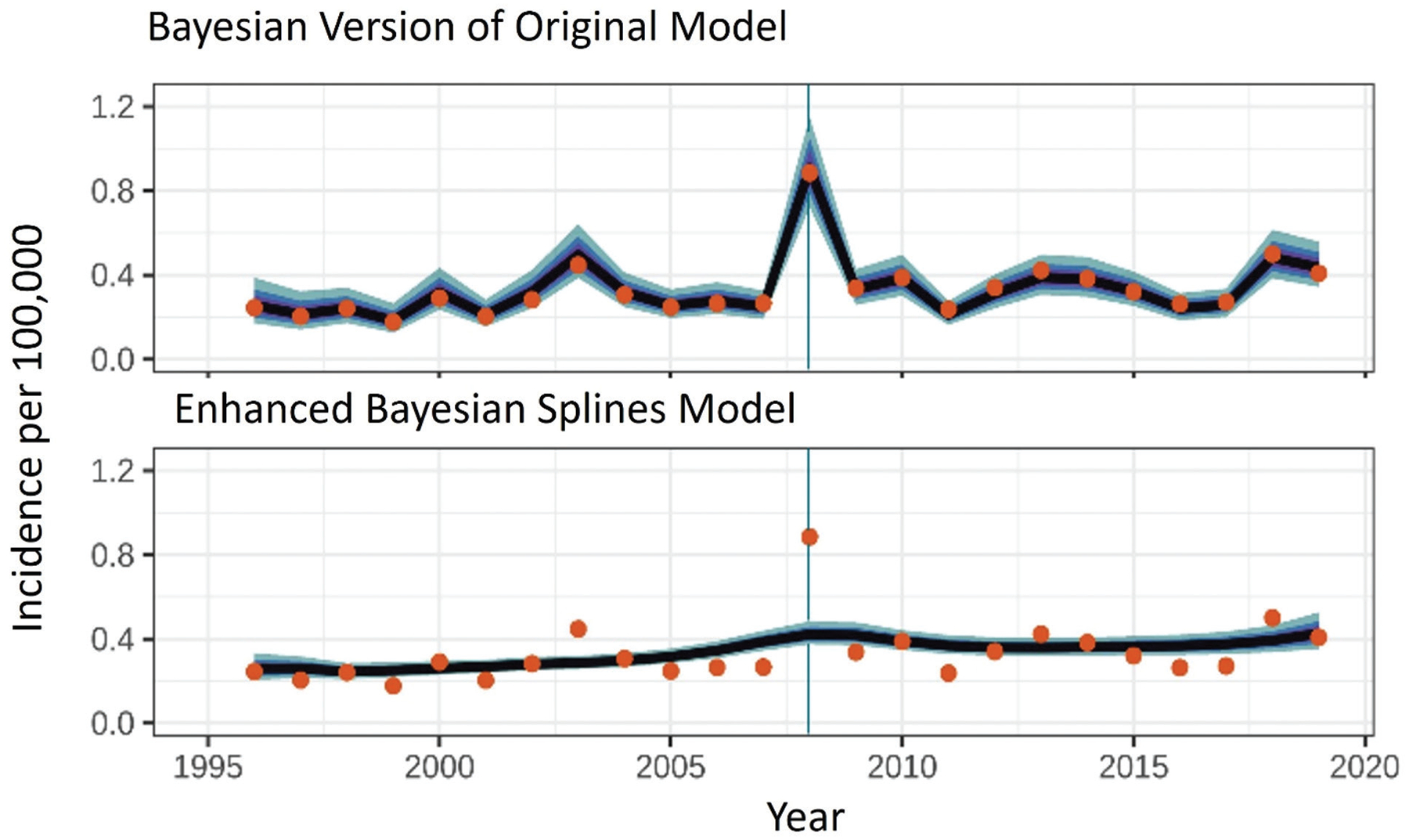
Actual (**dots**) and median estimates (line) of *Salmonella* Saintpaul illnesses per 100,000 in the FoodNet catchment. The shading represents the **50%**, **75%**, and **95%** credibility intervals (CrI) for these estimates. Note how sensitive the original model is to single-year aberrations compared to the splines model. Several spikes in the original graph correspond to well-publicized outbreaks linked to mangos in 2003, peppers in 2008, and cucumbers in 2013 [30–32]. While the original model was frequentist, a Bayesian version of that model was implemented here to ensure output was comparable to those of the new model.

**FIGURE 4 |:**
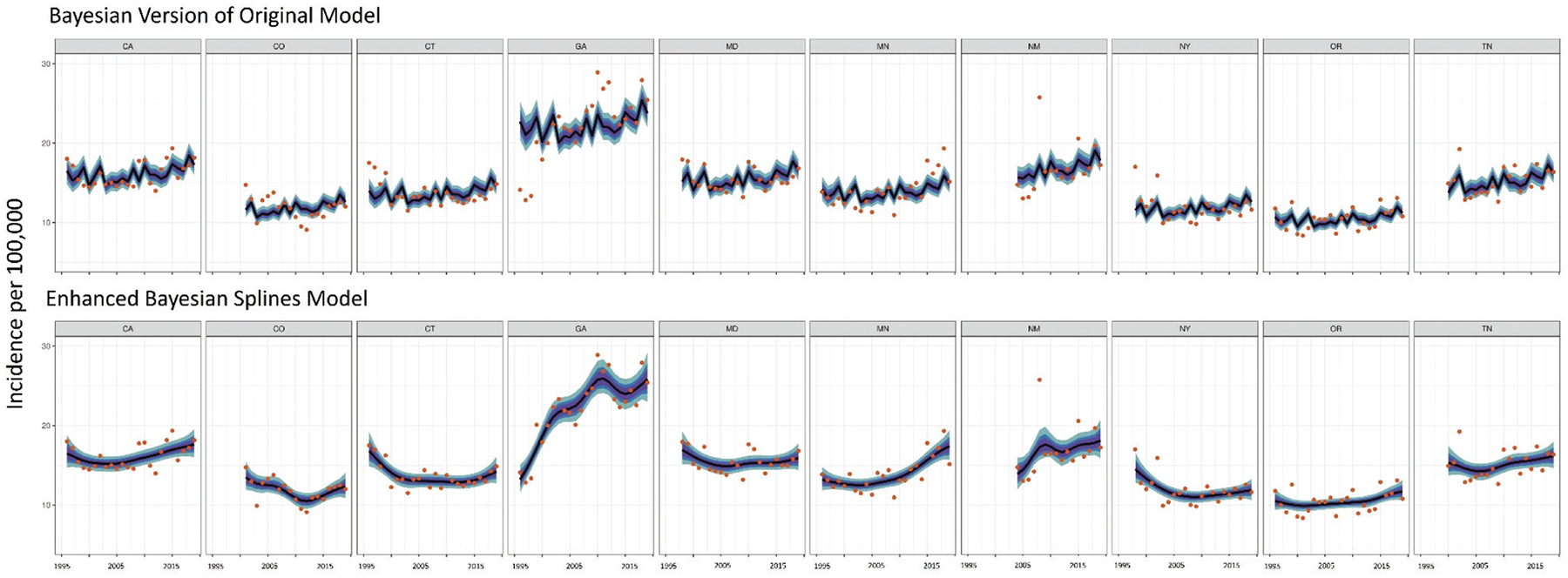
Actual (**dots**) and median estimates (line) of salmonellosis incidence per 100,000 based on the original model and the spline model for each FoodNet site. The shading represents the **50%**, **70%**, and **90%** CrI for these estimates. The original model forces the same trend onto each site, while the inclusion of a year–site interaction in the splines model allows for site-specific trends. As a result, the original model often under- or overestimates disease incidence for a given site in a given year; for instance, the original model substantially overestimated salmonellosis incidence in GA during 1996 through 2005. While the original model was frequentist, a Bayesian version of that model was implemented here to ensure output was comparable to those of the new model.

**FIGURE 5 |:**
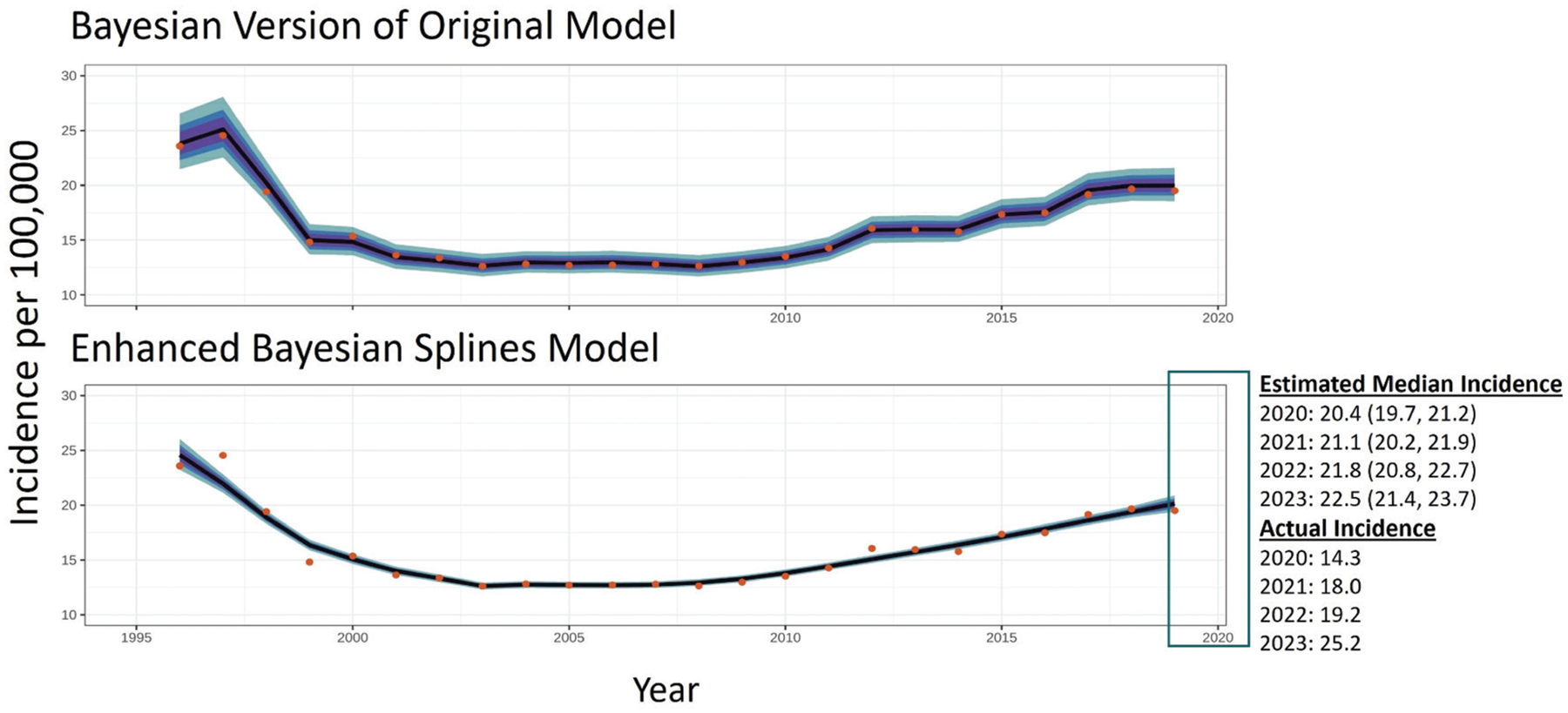
Actual (dots) and median estimates (line) of campylobacteriosis per 100,000 in the FoodNet catchment. The shading represents the **50%**, **70%**, and **90%** CrI for these estimates. Median annual incidence estimates were generated for 2020 through 2023 using the enhanced model. The discrepancy between estimated and observed incidence in 2020–2021 is likely attributable to reduced foodborne illness exposure and reporting during the COVID-19 pandemic, rather than to poor predictive performance. In contrast, the discrepancies observed for 2022 and 2023 likely reflect the increased temporal distance from the years used to train the model, highlighting greater uncertainty when extrapolating beyond the training window. Estimates could not be generated using the original model because the original model cannot be generalized to years not represented by the data (1996–2019). While the original model was frequentist, a Bayesian version of that model was implemented here to ensure output was comparable to those of the new model.

One of the chief improvements of the enhanced model is its utilization of a Bayesian framework, which allows the enhanced model to capture uncertainty more effectively through use of probabilistic parameter descriptions, particularly for years with limited or noisy data (compare CrIs in Figs 3–5). Past studies that directly compared Bayesian and frequentist performance further demonstrate this advantage [25,27,28]. One such study determined that Bayesian estimates were more consistent, exhibited less bias, and provided better coverage compared to estimates generated by the frequentist approaches considered [25].

Frequentist uncertainty measures, or CIs, provide information on long-term frequency properties but do not provide direct probabilistic insights. Interpretation of frequentist output is based on significance testing, or the probability of obtaining another dataset as, or more extreme than, the one collected based on an arbitrary threshold known as the *P*-value. In contrast, Bayesian approaches calculate the probability that a particular hypothesis is true given the observed data and a priori information. Because Bayesian analyses directly quantify the probability of specific outcomes, their outputs are generally considered more intuitive and less prone to misinterpretation than those from frequentist analyses [33,34]. A common misconception is that a frequentist 95% CI represents the range with a 95% probability of containing the true parameter value. In reality, this interpretation aligns with a Bayesian 95% CrI. Specifically, a 95% CrI is defined as the central region of the posterior distribution encompassing 95% of the estimated parameter values, whereas the frequentist CI reflects how often the interval would contain the true value if sampling were repeated many times from the same population and does not support probability statements. Thus, the shift from a frequentist to a Bayesian framework results in more consistent and less biased uncertainty estimates, reduces the potential for misinterpretation, and increases the model’s utility for science communication and translation.

The enhanced model is also better suited to fitting trends for specific sub-populations defined by multiple levels of stratification. Due to improved uncertainty estimation, Bayesian methods are generally more efficient and can produce reliable estimates even when data are highly stratified and sample sizes within each stratum are small. In contrast, frequentist analyses rely on the central limit theorem and the law of large numbers, so their performance declines as sample sizes decrease. The Bayesian framework, in contrast to the original model, facilitates adapting or extending the enhanced model [25,35]. Completed and ongoing projects are already leveraging this to better understand foodborne and enteric illness trends for specific populations of interest and case characteristics. For example, the enhanced model has been adapted to separately model trends for domestically acquired and international travel-associated illnesses, and for illnesses diagnosed using culture and culture-independent methods [5,10]. Because these and other studies [5,10,36,37] identified significantly different trends in illnesses diagnosed by culture and culture-independent methods, future extensions of the enhanced model will explicitly incorporate trends for illnesses diagnosed using different methods. Because increasing model complexity will increase the computational costs of the enhanced model, R packages that use alternative approximation approaches are being explored [e.g., R-INLA; [38]].

### The enhanced model better captures non-linearity in trends

Year was treated as categorical in the original model because it offered an intuitive, easy-to-implement way to address non-linearity and changes in catchment size over time. However, discretization of continuous variables comes with a loss of information and, often, performance [39–43]. Time is inherently unidirectional; however, categorizing temporal variables obscures both this unidirectionality and the relationships between time points. For example, the reported incidence of yersinosis in the United States was historically under-reported due to diagnostic challenges [37]. Following the widespread adoption of culture-independent diagnostic tests (CIDTs) in the 2010s, incidence increased sharply [5,37]. Consequently, incidence in consecutive pre-CIDT years, such as 2008 and 2009, was similar, but very different from incidence in post-CIDT years, such as 2018 and 2019 [5]. When time is treated as categorical, the model loses the information that 2009 follows 2008 and treats all years as equidistant, ignoring the true temporal relationships and forcing an unrealistic stepwise structure onto trend estimates. Moreover, when time is categorized, estimates for each year are only meaningful in the context of a pre-specified reference year, which forces an unrealistic step relationship onto trend estimates [Figs 3–5; [43–45]]. Due to these limitations, the enhanced model treated year as continuous.

Trends over time are also often non-linear, especially if the population or phenomenon being surveilled changes over time (e.g., due to catchment expansion, changing demographics, changing laboratory practices, changes in pathogen virulence or transmission). In these situations, including year as a linear covariate is also inappropriate and associated with loss of information [46]. Instead, statisticians often advocate the use of methods that preserve non-linearity while avoiding the pitfalls of categorization [47]. By using splines regression, the enhanced FoodNet model preserves the inherent non-linearity in disease trends over time. Splines regression is a smoothing approach that also helps remove signals from random noise (e.g., due to single-year events). Consequently, models that apply a spline parameter to non-linear continuous covariates often outperform those that treat the covariate as linear or categorize it [44,48]. The utility of splines for more accurately capturing long-term trends is demonstrated by the output of the original and enhanced model for *Salmonella* Saintpaul (Fig 3). The trend estimated by the original model is generally coarser and more variable (more and higher peaks than the trend estimated by the enhanced model). This is because the original model forces a step relationship onto year through categorization and is sensitive to random noise and single-year perturbations. In contrast, the use of splines in the enhanced model smooths over these signals and captures gradual changes in incidence over years. In 2007, there was a large multistate outbreak of *S*. Saintpaul infections linked to the consumption of contaminated peppers that sickened 1,442 people across the United States [49]. Note how sensitive the original model was to this single-year event, while the enhanced model shows a more smoothed trend that better reflects the incidence of *S*. Saintpaul in the FoodNet catchment over time. Indeed, based on the models’ R2 values, the enhanced model explained 12% more of the variation in observed *S*. Saintpaul incidence than the original model. This improvement reflects a materially better ability to separate true temporal patterns from random noise, increasing confidence in the resulting trend estimates.

Due to increased flexibility provided by splines, the enhanced model can also be more easily adapted to address new and emerging public health questions than the original model. For instance, when working with public health data, there are often events that result in missing, noisy, or biased data. Splines models can be used to generate estimates for years or sites with missing data after excluding them from model training. Multiple studies suggest that the incidence of reported foodborne illnesses decreased during the COVID-19 pandemic [7,9]. The enhanced model can be used to interpolate estimated incidence in 2020 and predict what the incidence would have been if the COVID-19 pandemic had not occurred. Such interpolations are not possible with the original model, because year is treated as a categorical variable and estimates can only be generated for categories present in the training data. While splines are mainly intended for interpolation (generating predictions within the range of training data), they can be cautiously used for nowcasting incidence for years close to the period represented by the training data. Because uncertainty in nowcasting estimates increases as you move further in time from the years represented by the training data, the most reliable estimates will be for the years immediately preceding and after the training period. Fig 5 shows how the enhanced model was able to estimate incidence for years outside the range used to train the model. A potentially valuable extension of the current study would be to validate specific time intervals during which nowcasting estimates closely align with actual incidence rates.

### By generating site-specific trend estimates, the enhanced model offers more reliable insights into national trends

The enhanced model better captures distinct trends within FoodNet sites than the original model. Because the original model forced the same trend onto each site, trends were largely driven by populous southeastern sites (e.g., GA, TN, MD). As a result, the model was not useful for generating site-specific estimates and did not fully represent trends in other U.S. regions or nationally. This is demonstrated by Fig 4, which shows salmonellosis estimates from the original and enhanced trends model for each FoodNet site compared to the actual incidence each year during 1996–2019. Because the same trend is forced onto all 10 sites, the original model frequently under- or overestimates incidence. For example, the 95% CI for estimated incidence in CO does not overlap actual incidence in CO for 43% of the time (N = 10). Even for populous sites, such as GA, the original model estimates do not include actual incidence 52% of the time (N = 12 years) with the model drastically over-estimating incidence in GA during 1996–2001. By including an interaction between year and site, the enhanced model overcomes this limitation and fits a separate spline for each FoodNet site. The 95% CrI for estimated incidence in CO overlaps actual incidence in CO 91% of the time (N = 21 years), while the 95% CrI for GA overlaps actual incidence in GA 78% (N = 18) of the time. By incorporating an interaction between site and year, the enhanced model better captures differing signals across the United States. The enhanced model output is therefore more likely to reflect and give insights into both site-specific and national trends than the original model.

### Next steps for continuous improvement and study limitations

When it was first implemented, the original model was incredibly useful as it allowed FoodNet to leverage the full scope of the FoodNet data. The original model was the optimal model that could be developed given the resources available when it was developed [6]. In 2001, it would not have been feasible to develop a Bayesian splines model because the computational resources and statistical software that underpin the enhanced model did not yet exist [6]. The enhanced model merely represents an update to the original model using computational and statistical resources that have emerged since 2001.

Just as the enhanced model builds on the original model, the enhanced model should be viewed as a foundation for future refinement rather than as a final, static model. For example, FoodNet has more granular data available than the model is currently using. This is because the algorithm underpinning the model (MCMC) is computationally intense and takes orders of magnitude longer to generate estimates using county compared to site-level data. A logical next step for enhancing the model would be to implement a less computationally intensive approximation method, such as R-INLA. This would enable the generation of more granular estimates in the near-real time using standard laptops instead of requiring high-performance computing environments. FoodNet is the United States’ sentinel surveillance system for foodborne illness and generates the most up-to-date, complete, and highest-resolution data on foodborne and enteric diseases in the United States. Making the enhanced model less computationally demanding will increase the utility of these data.

Once the enhanced model has been updated to reduce computational burden, countless follow-on enhancements are possible. Surveillance data are inherently spatial, and trends in adjacent counties are likely to be more similar than trends in non-adjacent counties. A past campylobacteriosis study that compared alternative parameterizations (i.e., by including additional demographic and temporal covariates) of the original model [50] noted their models did not sufficiently capture variation due to unmeasured spatial relationships in the data and recommended that future efforts to improve the original FoodNet trends model explore ways of doing so. Once we can fit county-level models, we can incorporate these spatial relationships into the model to improve estimates. Reducing the computational burden associated with running the trends model opens countless other possibilities for improving the enhanced model as well. For instance, past studies have shown that changing diagnostic practices are drastically affecting foodborne and enteric disease epidemiology, including incidence trends [e.g., [5,37]]. However, new diagnostic methods are being differentially adopted by labs serving different communities. Because new diagnostic methods can detect illnesses that would have previously gone undiagnosed, disentangling signals due to increased use of these methods from true increases in incidence is complicating interpretation of trends [5,37]. Incorporating a spline of the year with an interaction between the diagnostic method and site (or county) would allow us to, at least partially, overcome this complication.

In considering potential improvements to the enhanced model, it is important to consider the model’s temporal resolution. The original and enhanced FoodNet trends models both generate annual estimates. However, past studies have reported substantial intra-annual variation in foodborne and enteric disease incidence due to intra-annual changes in human behavior, pathogen shedding by non-human hosts, and environmental conditions, among other factors [50–58]. While the present study does not account for intra-annual variation, future efforts to enhance the FoodNet trends model should consider ways to capture these intra-annual signals. Such efforts will require careful consideration to ensure non-linearity of annual and intra-annual trends is preserved. Indeed, as future iterations of the trend model increase in complexity, it may be necessary to change the model distribution (e.g., to account for increases in structural zeros), or to develop separate models for different FoodNet-monitored pathogens [50].

Lastly, the enhanced model currently uses the default prior settings in *brms*, which are designed to be weakly informative or flat, allowing the data to drive inference while maintaining model stability and generating results comparable to frequentist approaches [33]. Specifically, the model employed a Student-t prior [*student_t*(3, −8.84, 2.5)] on the intercept, Student-t priors [*student_t* (3, 0, 2.5)] on standard deviation parameters, and an inverse-gamma prior [*inv_gamma*(0.4, 0.3)] on the shape parameter. Flat (uninformative) priors were specified for all regression coefficients, including site effects and their interactions with year. As a result, all possible values of the regression coefficients were considered equally likely, and no prior expectations were imposed. This decision was made to ensure comparability with the original frequentist methods. However, this approach has limitations. For example, incorporating weakly informative or informative priors could reduce computational time by constraining parameter spaces. However, this would require pathogen-specific specification, which the pipeline is not currently structured to accommodate. Future iterations of the enhanced model could address these limitations by conducting a sensitivity analysis comparing flat, weakly informative, and informative priors, and by exploring mechanisms for integrating pathogen-specific prior knowledge into the modeling pipeline.

## CONCLUSIONS

The new Bayesian FoodNet trends model builds on the previous frequentist model—both are negative binomial models with a log link function and a log offset for the population of each site each year. The new model reduces sensitivity to single-year aberrations (e.g., outbreaks), accounts for site-specific trends, and reduces biases toward more populous sites. It is also better able to capture uncertainty in model estimates, and can estimate data for years in which data are not available. By improving and publishing a pipeline for implementing the new trends model, FoodNet data will be more readily available and interpretable. Additional enhancements to the trends model will further facilitate the use of FoodNet data and make FoodNet data more available to inform public health action. Although the present study focuses on methodological performance rather than applications, future research could evaluate how enhanced trend estimates may support outbreak communication and guide intervention planning.

## Figures and Tables

**TABLE 1 |:** Differences between the original model described by Henao et al. [6] and the enhanced model.

	Original model	Enhanced model
Framework	Frequentist^a^	Bayesian
Dependent on significance testing	Yes	No
Uncertainty measures	95% confidence intervals	95% credibility intervals
Spatial considerations		
Unit of analysis	County-year	State
Bias	Toward more populous states	Not biased by site population
Site-specific trends	Same trend for all states	Site-specific trends
Trends		
Year	Categorical	Continuous
Non-linearity	Captured by treating year as categorical	Uses splines to capture non-linearity
Unidirectional nature of time	Not accounted for	Accounted for
Nowcasting	No	Yes

a The results presented here are for a modified version of the original model that used a Bayesian as opposed to frequentist framework. This model was fit using the default weakly informative priors in *brms*. These results were comparable to the output of the frequentist version of the original model.

## Data Availability

Data are available using the formal data request process described https://www.cdc.gov/foodnet/data/index.html/.

## Highlights

- FoodNet monitors trends in foodborne illnesses across 10 U.S. sites.
- The original FoodNet model was frequentist and had several limitations (e.g., sensitivity to single-year anomalies; bias toward trends in more populous surveillance sites).
- The enhanced model uses a Bayesian splines-based approach with a site–year interaction to capture non-linear, site-specific trends more accurately.
- The new framework improves uncertainty estimation, increases robustness to noisy or missing data, and supports near-term nowcasting.
- This paper makes a reproducible pipeline for implementing the enhanced model code, improving transparency and enabling adaptation to other datasets or population groups.
